# Safety and efficacy of L-Glutamine in reducing the frequency of acute complications among patients with sickle cell disease: A randomized controlled study

**DOI:** 10.1007/s00277-024-05877-8

**Published:** 2024-07-19

**Authors:** Fatma Soliman Elsayed Ebeid, Nihal Hussien Aly, Naglaa Mohammed Shaheen, Samah Mohammed Ahmed Abdellatif, Ahmed Ashraf Mahmoud Okba, Nada Ayman Gad, Sara Mostafa Makkeyah

**Affiliations:** 1 Pediatric Hematology Oncology and BMT Department, Faculty of Medicine, Ain Shams University, Cairo, Egypt; 2 Faculty of Medicine, Ain Shams University Research Institute-Clinical Research Center (MASRI-CRC), Cairo, Egypt; 3 Misr’s Children Hospital, Health Insurance Organization, Cairo, Egypt; 4 Radio-diagnosis Department, Faculty of Medicine, Ain Shams University, Cairo, Egypt; 5 Pediatric Department, Egyptian Atomic Energy Authority, National Centre for Radiation Research and Technology, Cairo, Egypt

**Keywords:** L-Glutamine, Sickle Cell Disease, Vaso-occlusive crisis, Children, Egypt

## Abstract

To evaluate the safety and efficacy of L-glutamine in reducing vaso-occlusive crisis (VOC) and improving cerebral arterial blood flow in children with sickle cell disease (SCD). This is an interventional randomized controlled trial that recruited sixty SCD patients, aged 9.2 ± 3.7 years, who had at least two VOCs during the last 12 months and on a stable dose of hydroxyurea. They were randomly assigned in a 1:1 ratio to receive glutamine (0.3 gm/kg/dose/12h) orally for 24 weeks or the standard of care (SOC). All patients had VOCs in the last year > 3, those on glutamine had a higher number of VOCs and hospitalization for VOC in the last year. There was a decreasing trend in the number, severity, and hospitalization of VOC and a significantly lower cumulative number of VOCs and hospitalizations in the glutamine group than in SOC (p = 0.008, p < 0.001 respectively). Time-averaged mean maximum velocity for the glutamine group had a marginal increase in both middle cerebral arteries, all values remained normal within a normal range, and in both internal carotid arteries, values increased from abnormally low to normal ranges at week 24. Glutamine reduced the number of VOCs and severity and may have a potentially favorable impact on the cerebral arterial flow velocities.

**Supplementary Information:**

The online version contains supplementary material available at 10.1007/s00277-024-05877-8.

## Introduction

Sickle cell disease (SCD) is one of the most common severe monogenic disorders rooted in a single base-pair point mutation of the hemoglobin β-chain resulting in an abnormal hemoglobin S (HbS) [1]. Patients with SCD suffer from unpredictable recurrent episodes of acute pain due to vaso-occlusive crisis (VOC) which may require hospitalization or from chronic persistent pain. This pain usually begins in infancy and continues throughout life, necessitating preventive rather than treatment measures [2].

The pathobiology of SCD is a vicious cycle of four major processes including HbS polymerization, impaired biorheology and increased adhesion-mediated vaso-occlusion, hemolysis-mediated endothelial dysfunction, and rigorous activation of sterile inflammation. These molecular and cellular processes synergize to promote both acute and chronic pain and result in end-organ injury and failure in SCD [3]. Although recurrent VOCs cause vital morbidity to those with SCD, however, morbidities can occur even in the absence of overt painful VOCs because of ongoing cumulative tissue injury [4]. Moreover, the unpredictable nature of this pain necessitates the use of opioids which is associated with several side effects, little is specific in the context of SCD, including hyperalgesia, tolerance, and addiction [5].

L-glutamine is an essential amino acid for the synthesis of the pyridines for nucleotides, including nicotinamide adenine dinucleotide (NAD) and glutathione, in addition to glutamate, especially during oxidative stress exposure. The sickle red blood cells (RBCs) had a lower NADH: [NAD^+^ + NADH] (redox) ratio than in normal RBCs which is related to oxidative stress, so the availability of L- glutamine is important in SCD [6]. In 2017, L-glutamine oral powder was approved by the U.S. Food and Drug Administration (FDA) to reduce SCD crisis episodes especially the VOCs. It can cause common non-serious adverse events like nausea, vomiting, stomach pain, gases, swelling in hands or feet, muscle or joint pain, back pain, headache, dizziness, tired feeling, mild skin rash or itching, dry mouth, runny nose, or increased sweating [7].

Our primary objectives were to assess the safety and efficacy of L-glutamine in patients with SCD in reducing the frequency of the occurrence of acute painful episodes from baseline to week 24 via calculating the percent change in the frequency of VOC. The secondary objectives were to determine the effect of L-glutamine on the percentage of change in transcranial Doppler (TCD)- time-averaged mean maximum velocity (TAMMV)- arterial cerebral blood flow, on the improvement of health-related quality of life (HRQOL) in patients with SCD from baseline to week 24, other exploratory objectives were to determine the changes in hematologic parameters and measures of hemolysis from baseline through week 24. The primary efficacy endpoint was the cumulative number of VOCs at 24 weeks, and the secondary endpoint was the change in TCD-TAMV in arterial cerebral blood flow at 24 weeks. The primary safety endpoint was the development of adverse events such as bleeding tendency, and significant impairment of liver or kidney functions.

## Subjects and methods

This was a prospective phase IV interventional open-label randomized controlled investigator-initiated clinical trial. Using the PASS 15 program for sample size calculation, setting power at 80%, and alpha error at 5%, based on the result of a pilot study, we assumed an effect size difference equals 0.8 between the two groups regarding the mean frequency of the occurrence of the acute painful episode, based on this assumption a sample size of at least 30 patients per group was used. Consequently, sixty children and adolescents with SCD were recruited from the Hematology clinic at Ain Shams University Children’s Hospital during the period from the 4th of January 2022 through the 3rd of January 2023. The study was conducted after approval of the Ethical Committee of Ain Shams University Hospitals under acceptance number FMASU M D 199/ 2021 and was registered in clinicaltrials.gov, identifier number NCT05371184. An informed consent and assent were obtained from each patient and control (whenever applicable) and their legal guardians before enrolment in the study. All procedures were performed per the ethical standards of the institutional research committee under the Helsinki Declaration 2013.

### Study population

Eligible participants were children and adolescents aged from five to eighteen years old, diagnosed with SCD by hemoglobin electrophoresis. Age eligibility was based on the FDA approval of l-glutamine oral powder in children aged 5 years and older [8]. They should have at least two documented VOCs with no upper limit during the previous year, where the VOC was defined as pain leading to treatment with a parenterally administered narcotic or non-steroidal anti-inflammatory in the emergency department (ED) or outpatient treatment centre or during hospitalization. For participants taking hydroxyurea (HU), the dose of HU (mg) per kg should be stable for at least 90 days before enrolment, and with no anticipated need for dose adjustments (other than weight-based). For participants not taking HU, there should be no anticipated need for initiation of HU in the opinion of the investigator. Diagnosis of SCD was made based on clinical picture, complete blood count (CBC), reticulocyte count, markers of hemolysis as well as hemoglobin analysis by high-performance liquid chromatography (HPLC) [9]. Patients with clinically significant renal, or liver disease (prothrombin-time international normalized ratio (INR) higher than 2.0, or serum albumin level of less than 3.0 g/dL), and those who received any blood products within 3 weeks before enrolment in the study were excluded.

### Intervention

**Fig. 1:**
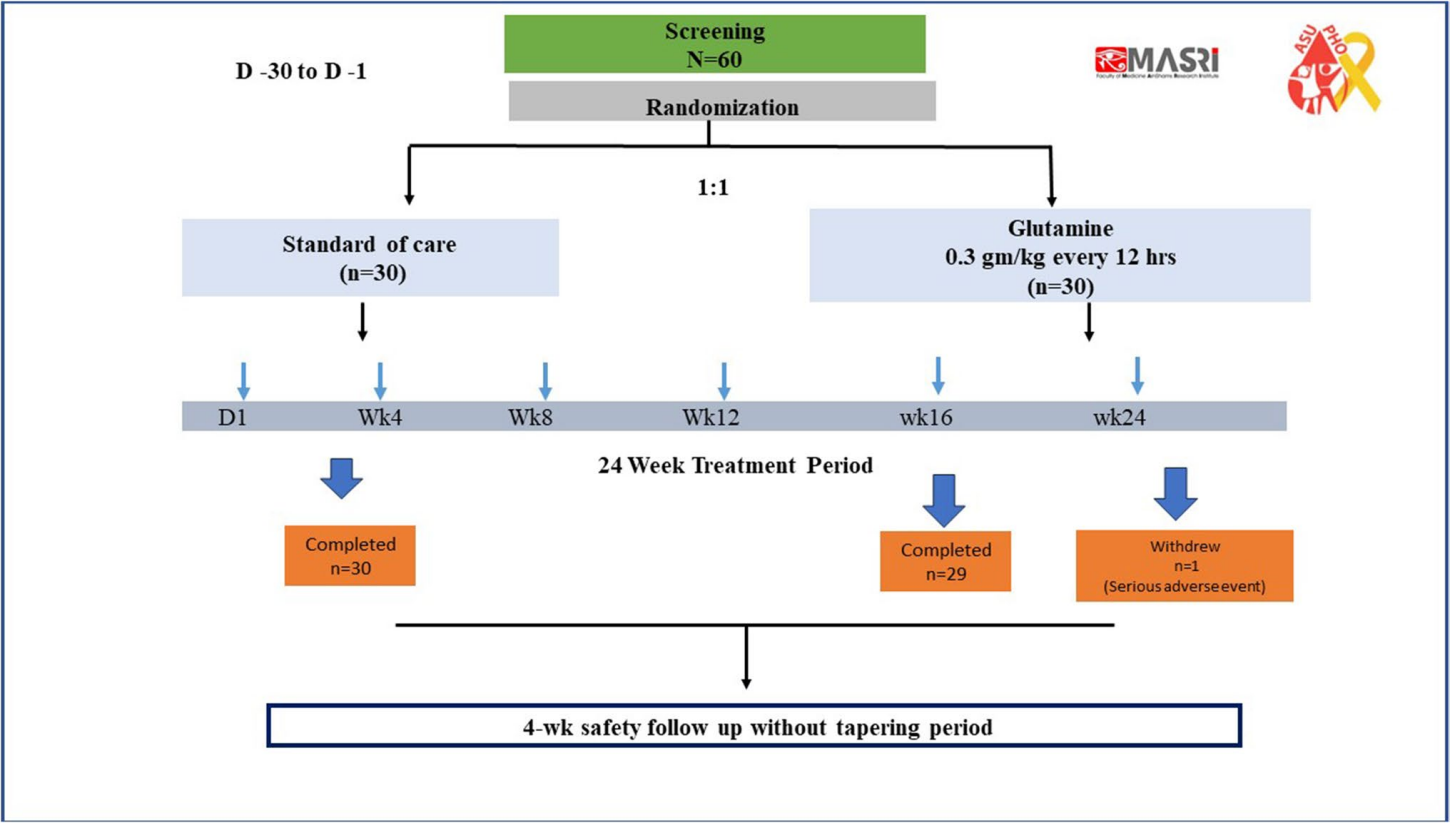
Flow chart for patients’ disposition

This is an investigator-initiated single-center prospective phase IV open-label interventional randomized controlled trial at Ain Shams Pediatric Hematology Unit. This research did not receive any specific grant from funding agencies in the public, commercial, or not-for-profit sectors. Sixty participants were randomly assigned in a 1:1 ratio to receive glutamine for 24 weeks or the standard of care (SOC) without glutamine intake (Fig. 1). No placebo was used in this study owing to the technical difficulty of making a placebo of the same chemical and physical criteria at our institute consequently L glutamine was used as an add-on therapy to the SOC. The thirty patients in the interventional arm received l-glutamine as a powder that was mixed with water or any non-heated beverage or with any non-heated food such as yogurt, applesauce, or cereal immediately before ingestion orally twice a day [10], dosed based on body weight (0.3 g per kilogram per dose) and was adjusted in increments of 5 g up to a maximum of 15 g/dose with an upper limit of 30 g per day. Patients were given verbal instructions for self-administration of the study medication and written instructions were also included on the consent form. Patients were instructed that the study drug should be taken between 6 and 9 am and again between 6 and 9 pm. The main side effects of l-glutamine reported in the SCD clinical trials were constipation, nausea, headache, abdominal pain, cough, extremity pain, back pain, and chest pain [11].

### Study tools

#### The detailed history

All patients were subjected to the detailed history through direct interviews of the subject and/or caregiver as well as collecting the data from the filling system including demographic characteristics, history of VOCs including the number of VOCs in the last year, its frequency per month, type and route of analgesics received at home and during hospitalization, hospitalization history, the severity of VOC, duration of admission in days, as well as the history of other complications, comorbidities, and operations. Detailed blood transfusion history (age of start transfusion, pre-transfusion Hb, simple and exchange transfusion, frequency per year and the complications as regards alloimmunization and infections), drug history including HU (age of start in years, maximum dose/kg/day, duration, compliance, and side effects), iron chelation (age of start in years, type, dose, duration, compliance, and side effects) was recorded.

#### Assessment of health-related quality of life (QoL)

HRQoL at the beginning and the end of the study using the Peds QL™ 3.0 SCD Module, Arabic version questionnaire for Children from 5–7, 8–12 years, Parents of children aged 5–7 and 8–12 years composed from 42 items comprising 9 dimensions. The questions included “feeling of pain, effects of pain, dealing with pain, feeling of anxiety, fearing, feeling of suffering from complications, feeling problems due to suffering sickle cell, problems of treatment and communications with others.” Their answers ranged from never a problem, problem sometimes, or problem many times [12].

#### Detailed physical examination

This includes weight in kg (standard deviation), height in cm (standard deviation), and body mass index (standard deviation). Growth stunting is identified by comparing measurements of children's heights and weight to the World Health Organization (WHO) 2006 growth reference population: children who fall below the fifth percentile of the reference population in height and weight for age are defined as stunted, regardless of the reason [1]. Sexual maturity rating using Tanner score [13] was assessed, where delayed puberty and hypogonadism were defined as an absence of sexual characteristics in girls till the age of 13 years and a testicular volume < 4 ml in boys till the age of 14 years [14], also detailed cardiac, chest, abdominal, and neurological examination were performed.

#### Laboratory

Baseline hemoglobin electrophoresis (HPLC) performed using D-10 (Bio-Rad, Marnes La Coquette, France) for initial Hb F levels, CBC with reticulocyte count using Sysmex XT-1800i (Sysmex, Kobe, Japan), markers of hemolysis in form of total serum and indirect bilirubin, alanine aminotransferase (ALT), aspartate transaminase (AST*)*, and serum creatinine using Cobas Integra 800 (Roche Diagnostics, Mannheim, Germany) were performed before starting treatment with l-glutamine and on monthly bases through the 24 weeks, while serum ferritin level was measured at entry of the study on Immulite 1000 analyzer (Siemens Healthcare Diagnostics, Marburg, Germany).

#### Transcranial doppler (TCD)

TCD was performed for all participants (Figure S1) by a certified trained sonographer with the calculation of the TAMMV in the right and left MCA, right and left ACA, and right and left ICA at three-time points: before the start of treatment, after 12 weeks (three months) of glutamine, and at the end of the study in both study arms. The TAMMV was considered very low if less than 70 cm/s, normal if less than 170 cm/s, conditional if between 170 – 200 cm/s, and abnormal if above 200 cm/s [15].

#### Monthly clinic visits

Patients have been examined once monthly to answer questions designed to monitor compliance and adverse effects while the patient and/or parent completed a daily compliance diary, history of crises including the time to the first painful crisis after starting the L- glutamine and frequencies, severity with the need for hospitalization. A committee responsible for the justification of whether the event is considered VOC or not was formed by the investigators (Fatma Ebeid, Sara Mekkya, Nihal Hussien). QoL was assessed for all patients as well as their parents at the beginning and the end of the study, whereas TCD was evaluated for all participants at the beginning, after 12 weeks (after three months of treatment with glutamine), and at the end of the study.

#### Statistical methods

Demographic and clinical data will be obtained and recorded in a special registration form. Statistical data will be tabulated, and Statistical analysis is to be performed using Microsoft® Excel® version 2016 and SPSS® for Windows® version 27. Quantitative data will be presented as mean and standard deviation. An independent t-test will be used to compare the means of the two groups. Qualitative data will be presented as numbers and percentages. The chi-square test or Fisher exact test will be used to compare between two or more proportions. The two groups were compared first as regards the presence or absence of VOCs using the Chi-Square test, then the cumulative number of VOCs by the Mann–Whitney test. Univariate Odds ratio/Risk ratio will be calculated for each studied risk factor. The significance level was set at 0.05.

## Results

Sixty participants were recruited, their mean age was 9.2 ± 3.7 years, and they were 35 males and 25 females. Thirty SCD patients were recruited in each group either received glutamine or SOC, both groups were comparable as regards demographic data (Table 1). All patients in both arms had VOCs in the last year more than three, however patients on glutamine had a higher number of VOCs (P < 0.001) and hospitalization (P = 0.006) for VOC in the last year, but the VOC severity was comparable between the two groups at baseline (Table 2), in addition, they had higher hospitalization frequency for causes other than VOC. SCD complications illustrated in Table 1, all recruited participants had a history of hemolytic crisis, while. none of the patients in either group had a history of hepatic sequestration, intrahepatic cholestasis, HBV infection, manifest renal or cardiac complication, or leg ulcer.

**Table 1:** Comparison between the two studied groups regarding their demographic and disease characteristics

		Glutamine arm (Intervention group) n = 30	Standard of care arm (Control group) n = 30	P-value
Gender; n (%)	Females	15.0 (50.0)	10.0 (33.3)	0.190
	Males	15.0 (50.0)	20.0 (66.7)	
Age (Year); Mean ± SD		9.1 ± 3.5	9.4 ± 3.9	0.690
Positive Consanguinity; n (%)		14.0 (46.7)	15.0 (50.0)	0.796
Positive family history of SCD; n (%)		18.0 (60.0)	20.0 (66.7)	0.592
Age of diagnosis in months; Mean ± SD		20.2 ± 27.4	26.7 ± 22.6	0.316
Disease Duration (Years); Median (IQR)		7.0 (5.0—8.5)	6.3 (5.0—10.4)	0.739
Hb electrophoresis	HbA%; Median (IQR)	9.9 (0.0 – 22.0)	8.6 (0.0 – 20.0)	0.485
	HbA2%; Median (IQR)	3.0 (2.4—3.8)	3.2(2.7 – 4.0)	0.336
	HbF%; Median (IQR)	14.5 (10.1 – 24.0)	11.9 (8.3—17.3)	0.261
	HbS%; Median (IQR)	63.5 (60.0—73.5)	72.0 (62.5—77.2)	0.108
Type of sickle; n (%)	SB0%	3.0 (10.0)	1.0 (3.3)	0.285
	SS%	19.0 (63.0)	18.0 (60.0)	
	SB + %	6.0 (20.0)	10.0 (33.3)	
	SC%	0.0(0.0)	1.0 (3.3)	
	SO arab%	2.0 (6.7)	0.0 (0.0)	
Comorbidities	Sequestration crisis; n (%)	1 (3.3)	2 (6.7)	0.554
	Aplastic crisis; n (%)	5 (16.7)	0.0	NA
	Acute chest syndrome; n (%)	11 (36.7)	4 (13.3)	0.037
	Manifest Stroke; n (%)	0(0.0)	1 (3.3)	0.313
	Avascular necrosis of hip; n (%)	1 (3.3)	1 (3.3)	1.000
	Cholecystitis; n (%)	4 (13.3)	4 (13.3)	1.000
	Cholelithiasis; n (%)	4 (13.3)	1 (3.3)	0.161
	Splenectomy; n (%)	5 (16.7)	5 (16.7)	1.000
	Cholecystectomy; n (%)	4 (13.3)	5 (17.2)	0.676
	Others; n (%)	5(16.7)^*^	3 (10)^#^	0.448
Frequency of hospital admission for causes other than vaso occlusive crisis (VOC) /year; Median (IQR)		4 (2—6)	0 (0—2)	0.000
Duration of hospital admission for causes other than VOC/year (day); Median (IQR)		12 (6—20)	10 (5—15)	0.627
Hydroxyurea	Age of start (years); Median (IQR)	2 (2—2)	2 (1—4)	0.810
	Duration (year); Median (IQR)	6 (4—9)	6.5 (3—8)	0.705
Blood Transfusion	Age at first transfusion (year); Median (IQR)	1 (0.7—2)	2.5 (1—4.5)	0.005
	Pre transfusion HB mg\dl; Mean ± SD	6.2 ± 0.8	6.5 ± 1.1	0.158
	Simple transfusion frequency/year; Median (IQR)	6.5 (3—12)	4.5 (2—6)	0.008
	Exchange transfusion; n (%)	9(30)	5 (16.7)	0.222
Iron Chelation	Deferasirox	15 (50)	14 (46.7)	0.796
	Dose mg/kg/day; Mean ± SD (Range)	19.4 ± 4.15 (14 – 28)	22.57 ± 2.65 (20 – 28)	0.022
	Age at start in year; Mean ± SD (Range)	3.27 ± 1.67 (2 – 6)	2.71 ± 1.86 (2 – 8)	0.406
Pain killer; n (%)	Duration in year; Mean ± SD	5.8 ± 2.54	7.21 ± 3.72	0.240
	Paracetamol	11 (36.7)	8 (26.7)	0.001
	Non- steroidal anti inflammatory	13 (43.3)	3 (10.0)	
	Both	6 (20)	19 (63.3)	
Pain killer frequency; n (%)	On demand	22 (73.3)	30 (100)	0.002
	Regular	8 (26.7)	0 (0.0)	

Other complications in glutamine arm^*^ included portal vein thrombosis, splenic infarction, osteomyelitis, and two performed surgeries for tendon release and tonsillectomy and adenoidectomy. While in control group^#^ one patient had hypoparathyroidism and hypothyroidism, and the other patient had G6PD deficiency

**Table 2:** Comparison between the two studied groups regarding vaso-occlusion crisis parameters of the studied patients through the six months of study period

		Glutamine arm (intervention group) n = 30	Standard of care arm (Control group) n = 30	P-value
Baseline				
Number of vaso -occlusion crisis Last year; Median (IQR)(Range)		12.0 (7.0 – 20.0) (6.0 – 25.0)	7.0 (6.0 -10.0) (4.0 – 20.0)	< 0.001
Mean Duration of Vaso-occlusion(days); Mean ± SD (Range)		3.3 ± 1.2 (2.0 – 5.0)	3.7 ± 1.7 (2.0 – 10.0)	0.302
Severity of vaso ooclusion crisis; n (%)	Non-severe	18.0 (60.0)	11.0 (36.7)	0.071
	Severe	12.0 (40.0)	19.0 (63.3)	
Laboratory data				
Hemoglobin (g\dl); Mean ± SD (Range)		8.7 ± 1.5 (6.8—12.4)	8.7 ± 1.7 (6 – 13)	0.937
White blood cells (10^3^/UL); Median (IQR)(range)		8.5 (7.2—13.9) (4.2—27.6)	9.1 (6.3—13.9) (4.5 – 21)	0.802
Platelet(10^3^/UL); Median (IQR) (Range)		315.5 (226—395) (73 – 1076)	312.5 (240—501) (108 – 815)	0.802
Retics (%); Median (IQR) (Range)		4 (2.5—6.5) (2 – 14)	3 (2.2—4) (0.5—13.7)	0.068
Serum ferritin(ng/ml); Median (IQR) (Range)		557.5 (311—1538) (100 – 3756)	665 (260—2139) (100– 5000)	0.535
Total bilirubin (mg/dl); Mean ± SD (Range)		2..2 ± 1.1 (0.5—4.2)	2.3 ± 1.1 (0.6 – 5)	0.591
Direct bilirubin(mg/dl); Mean ± SD (Range)		0.4 ± 0.2 (0.1—0.9)	0.37 ± 0.21 (0.1—0.8)	0.779
Alanine aminotransferase (U/L); Median (IQR) (Range)		23.0 (19—30) (14 – 76)	23.4 (20—32) 16.7 – 81	0.515
Aspartae aminotransferase (U/L); Median (IQR) (Range)		37.4(28—45) (13 – 100)	44.5 (34—56) (22.4 – 103)	0.042
Urea(mg/dl); Mean ± SD (Range)		21.1 ± 5.7 (8—32.3)	19 ± 5.3 (11.7 – 33)	0.140
Creatinine(mg/dl); Mean ± SD (Range)		0.5 ± 0.2 (0.2—0.8)	0.4 ± 0.1 (0.2—0.6)	0.354
Sixth month of start of glutamine				
Vaso-occlusion crisis; n (%)		2 (6.7)	9 (30.0)	0.02
Number/ month; Median (IQR) (Range)		1 (1—1) (1 – 1)	1 (1—1) (1 – 1)	1
Mean duration(days); Mean ± SD (Range)		1 ± 0 (1 – 1)	2.6 ± 0.5(2 – 3)	0.001
Severity; n (%)	Non-severe	2 (100.0)	7 (77.8)	0.118
	Severe	0 (0.0)	2 (22.2)	
Hospitalization for vaso-ooclusion crisis; n (%)		0 (0.0)	4 (44.4)	0.461
Laboratory data				
Hemoglobin(g\dl); Mean ± SD (Range)		8.5 ± 0.7 (7—10.6)	9.01 ± 1.4 (6.9—12.8)	0.080
Platelet(10^3^/UL); Median (IQR) (Range)		280.5 (250—330) (95 – 880)	295 (248—350) (190 – 889)	0.344
White blood cells (10^3^/UL); Median (IQR) (Range)		7.9 (6.6—9) (4.5 – 78)	8.6 (7.2—16) (5.8—22.4)	0.203
Retics (%); Median (IQR) (Range)		2.7 (2.2—3.4) (0.8 – 26)	3.05 (2.2—4) (0.5 – 5)	0.124
Aspartae aminotransferase(U/L); Median (IQR) (Range)		30 (25—32) (19 – 39)	29.5 (19—44) (13 – 86)	0.870
Alanine aminotransferase (U/L); Median (IQR) (Range)		27 (24—30) (12 – 34)	20 (19—22.5) (14 – 29)	< 0.001
Total bilirubin(mg/dl); Mean ± SD (Range)		2.92 ± 4.22 (0.8 – 25)	2.94 ± 0.51 (2.2—3.8)	0.976
Urea (mg/dl); Mean ± SD (Range)		21.17 ± 2.15 (18 – 29)	32.3 ± 37.3(21 – 229)	0.108
Creatinine (mg/dl); Mean ± SD (Range)		0.4 ± 0.1 (0.3—0.6)	0.3 ± 0.1 (0.2—0.6)	0.002

Physical examination of patients revealed 13 (43.3%) in the glutamine arm and 14(63.3%) in the standard of care arm had hepatosplenomegaly, their median Tanner score (IQR) 1 (1–1) and 1 (1–2) for glutamine arm, the standard of care arm respectively (P = 0.007). Their weight SDS and BMI SDS were significantly lower glutamine arm (p = 0.001, 0.002) than in SOC.

All recruited children who received HU were compliant and on stable doses except at week 12, two patients in the glutamine arm had an increase in dose as they developed hemolytic anemia and one patient in SOC had an increase in dose of HU due to the severity of VOC during hospitalization. All patients received prophylactic folic acid to prevent megaloblastic anemia and cardio-protective l-carnitine as SOC.

All patients received a blood transfusion at a certain time point in their lives; those on glutamine started transfusion at a younger age, as regard complications one patient in each group had a HVC infection, and alloimmunization was reported in three patients in the glutamine arm, and two in SOC arm. Through the study, seven patients on glutamine received simple transfusion due to a drop in hemoglobin level, and three patients needed exchange, one of them had a history of splenic infarction, and the other two had exchange transfusion due to severe VOC in the previous year while fifteen patients in the SOC arm received simple transfusion due to drop of hemoglobin and four patients required exchange transfusion, due to severe VOC in the previous year. Fifteen patients on glutamine received iron chelation (deferasirox) all were compliant except one patient who developed thrombocytopenia. Fourteen patients on SOC received deferasirox and had no adverse effects except one patient who had elevated liver enzymes.

As regards the efficacy endpoint, at baseline, there was a higher number of VOC and hospitalizations due to VOC in the last year in glutamine than those in the SOC arm. On the follow-up (Table 2) there was a decrease in the number, severity, and hospitalization of VOC in the glutamine arm and there was a significantly lower cumulative number of VOCs in the glutamine group than SOC, the mean of the percent of change in the frequency of VOC was 83.33 ± 36.44 and lower cumulative number of hospitalizations (p < 0.001). As regards the hemoglobin and markers of hemolysis, there was no difference between the two groups except at week 12, the reticulocyte count was higher in those on SOC (P = 0.000).

**Fig. 2:**
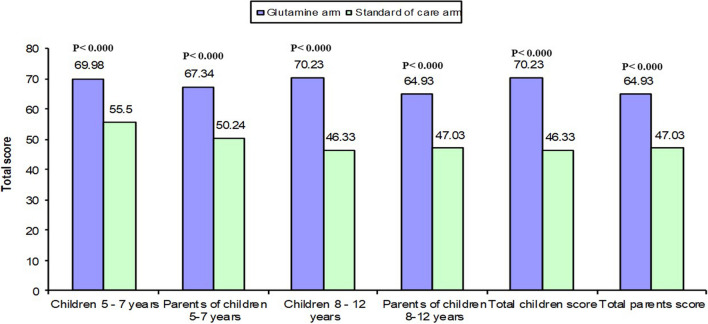
Total score of children and parents aged 5—7 years and 8–12 years at follow-up after six months of treatment of glutamine

**Fig. 3:**
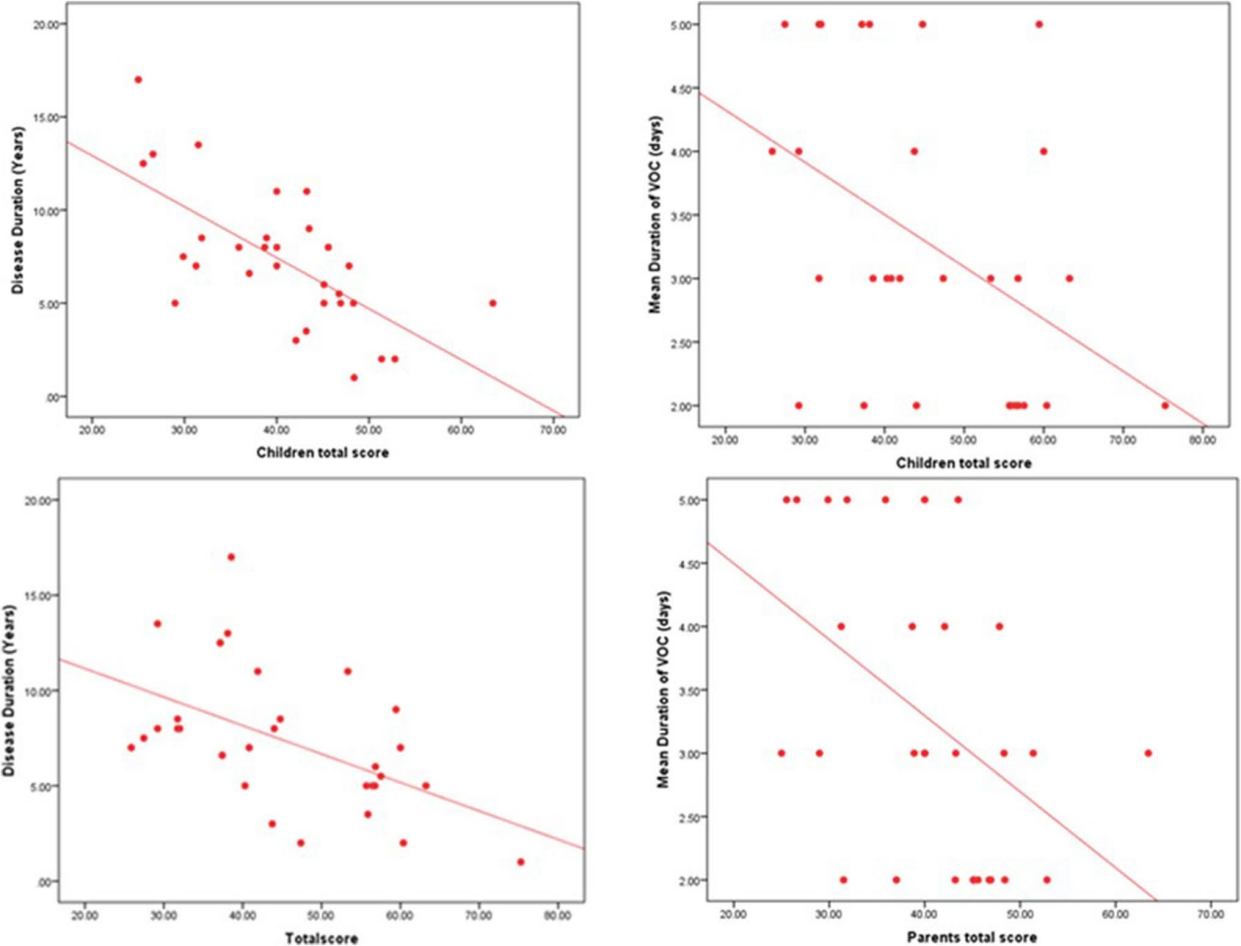
Correlation between children's total score with disease duration (years) and mean duration of VOC (days) (Above) and between parents' total score with disease duration (years) and mean duration of VOC (days)

As regards TCD parameters at baseline and at follow-up in the glutamine arm, illustrated in Table 3 and Figure S2, showed that there were four patients in the glutamine arm below 70cm/sec in the left MCA at baseline, dropped to three patients at week 12 and none at week 24, while no significant changes were recorded in the reading of TCD all through the time during the study period in SOC. Regarding the quality of life, patients in glutamine arm had significant improvement in pain, how to deal with pain, anxiety, anger, and treatment problems for children and parents of children aged 5–7 years and age 8–12 years as illustrated in Fig. 2 and 3 as well as Table 4 and S1.

**Table 3:** Comparison between the two studied groups regarding Transcranial Doppler parameters at baseline, after three months and after six months of follow up

TCD parameter	Base line		P-value	After three months		P-value	After six months		P- value
	Glutamine arm	Standard of care arm		Glutamine arm	Standard of care arm		Glutamine arm	Standard of care arm	
Rt Middle cerebral artery (cm/sec); Mean ± SD (Range)	113.9 ± 18.8 (80 – 150)	108.2 ± 27.1 (43 – 163)	0.520	113 .0 ± 17.8 (82 – 161)	117.3 ± 21.7 (72 – 150)	0.406	117.7 ± 13. 5(90 – 159)	117.3 ± 21.7 (72 – 150)	0.932
Lt Middle cerebral artery (cm/sec); Mean ± SD (Range)	108.2 ± 27.1 (43 – 163)	122.7 ± 25.8 (75 – 179)	0.038	110.7 ± 24.3 (65 – 159)	122.7 ± 25.0 (75 – 179)	0.069	116.1 ± 17.6(70 – 155)	122.7 ± 25.7 (75 –179)	0.252
RT anterior cerebral artery (cm/sec); Mean ± SD (Range)	106.7 ± 23.9 (40 – 156)	101.8 ± 24.2 (59 – 160)	0.437	109.1 ± 22.4 (57 – 158)	101.8 ± 24.2 (59 – 160)	0.237	111.2 ± 15.9 (60 – 145)	101.8 ± 24.2 (59 – 160)	0.082
LT anterior cerebral artery (cm/sec); Mean ± SD (Range)	111.5 ± 13.2 (89 – 145)	105.2 ± 24.5 (58 – 154)	0.214	109.3 ± 15.3 (78 – 133)	105.2 ± 50 (58 – 154)	0.438	113.7 ± 13.1(90 – 130)	105.2 ± 24.3 (58 – 154)	0.098
RT internal carotid artery (cm/sec); Mean ± SD (Range)	64.5 ± 21.2 (26 – 95)	54.2 ± 20.8 (31 – 106)	0.062	66.7 ± 18.5 (30 – 97)	53.7 ± 20.9 (31 – 106)	0.014	72.4 ± 15.6 (38 – 90)	53.4 ± 20.5 (31 – 106)	< 0.001
LT internal carotid artery (cm/sec); Mean ± SD (Range)	64.2 ± 18.5 (30 – 105)	5 7.4 ± 22.0 (25 – 114)	0.202	67.0 ± 15.7 (28 – 103)	58.9 ± 22.5 (25 – 114)	0.113	72.5 ± 13.4(30 – 95)	58.1 ± 21.8 (25 – 114)	0.003

**Table 4:** Comparison between the two studied groups regarding PedsQl (Acute version 3.0 Arabic Egypt) at baseline and follow up after six months

PedsQL (Acute Version 3.0 Arabic Egypt) Mean ± SD	Baseline		P-value	follow up after six months		P-value	Baseline/follow up	
	Intervention group n = 30	Control group n = 30		Intervention group n = 30	Control group n = 30		Intervention group P-value	Control group P-value
**Children aged 5—7 years**								
My pain	60.4 ± 11.4	54.9 ± 11.6	0.243	77.4 ± 5.5	54.9 ± 11.6	** < 0.001**	** < 0.001**	1.000
Effects of pain	49.3 ± 18.8	53.4 ± 12.4	0.513	71.3 ± 13.4	53.4 ± 12.4	**0.002**	**0.003**	1.000
How to deal with your pain	56.7 ± 20.6	54.9 ± 12.2	0.719	75 ± 15.1	54.3 ± 12.3	** < 0.001**	**0.020**	1.000
Anxiety problems with pain	48.6 ± 17.5	56.5 ± 6.2	0.132	63.4 ± 11.6	56.6 ± 6.8	**0.077**	**0.030**	1.000
Angry feeling	38.3 ± 18.0	45.7 ± 18.3	0.312	63.4 ± 11.5	45.7 ± 18.3	**0.008**	** < 0.001**	1.000
Treatment problems	66.9 ± 12.6	64.5 ± 9.2	0.577	76.2 ± 11.8	64.5 ± 9.2	**0.011**	0.091	1.000
Communications problems	61.7 ± 17.1	58.1 ± 11.5	0.533	63.3 ± 8.3	59.1 ± 12.2	0.314	0.755	0.336
Total score	54.5 ± 10.2	55.3 ± 5.7	0.805	69.9 ± 7.7	55.5 ± 5.8	** < 0.001**	** < 0.001**	0.336
**Parents of children**								
Feeling of pain	55.2 ± 12.5	55.2 ± 7.3	0.989	82.2 ± 6.2	55.2 ± 7.2	** < 0.001**	** < 0.001**	1.000
Effects of pain	45.2 ± 16.3	54.7 ± 11.6	0.095	73.6 ± 8.8	54.7 ± 11.6	** < 0.001**	** < 0.001**	1.000
Dealing with pain	44.2 ± 11.6	61.4 ± 5.3	**0.000**	73.3 ± 17.7	60 ± 0.0	**0.009**	** < 0.001**	0.336
Feeling of anxiety, fearing	40 .0 ± 9.2	40.6 ± 18.2	0.923	72 ± 13.8	40.5 ± 18.2	** < 0.001**	** < 0.001**	1.000
Feeling of suffering from complications	40.8 ± 12.4	37.14 ± 18.99	0.57	60 ± 0	37.1 ± 18.9	** < 0.001**	** < 0.001**	1.000
Feeling Problems due to suffering sickle cell	38.3 ± 11.2	37.1 ± 18.9	0.85	56.6 ± 7.7	37.1 ± 18.9	**0.003**	** < 0.001**	1.000
Problems of treatment	60.8 ± 17.5	59.1 ± 17.9	0.8	60.8 ± 2.8	59.1 ± 17.9	0.736	1.000	1.000
Communications with others	56.1 ± 10.8	58.1 ± 11.5	0.656	60 ± 0.0	58.1 ± 11.5	0.573	0.238	1.000
Total score	47.5 ± 6.5	50.4 ± 5.9	0.255	67.3 ± 4.2	50.2 ± 5.9	** < 0.001**	** < 0.001**	0.336
**Children aged 8—12 years**								
My pain	41.1 ± 12.6	39.4 ± 11.8	0.695	78.7 ± 2.8	39.5 ± 11.8	** < 0.001**	** < 0.001**	0.333
Effects of pain	28.6 ± 12.8	45.75 ± 12.22	**0.000**	62.2 ± 6.2	45.4 ± 12.5	** < 0.001**	** < 0.001**	0.333
How to deal with your pain	40.0 ± 19.4	43.7 ± 18.2	0.567	72.2 ± 15.5	46.25 ± 17.4	** < 0.001**	** < 0.001**	0.333
Anxiety problems with pain	29.3 ± 16.3	36.5 ± 15.8	0.204	63.5 ± 7.3	36.7 ± 15.8	** < 0.001**	** < 0.001**	0.333
Angry feeling	38.8 ± 14.5	38.75 ± 17.08	0.98	76.6 ± 10.3	41.5 ± 19.9	** < 0.001**	** < 0.001**	0.333
Treatment problems	48.3 ± 17.5	52.3 ± 19.6	0.54	69.2 ± 5.6	57.3 ± 21.1	**0.027**	** < 0.001**	0.333
Communications problems	52.6 ± 14.8	52.9 ± 16.4	0.952	68.9 ± 15.2	57.9 ± 17.8	0.061	**0.003**	0.333
Total score	39.8 ± 10.9)	44.2 ± 6.9	0.182	70.2 ± 6.4	46.3 ± 8.4	** < 0.001**	** < 0.001**	0.336
**Parents of children**								
Feeling of pain	36.9 ± 14.9	47.4 ± 11.1	**0.028**	70.9 ± 4.9	47.4 ± 11.1	** < 0.001**	** < 0.001**	1.000
Effects of pain	32.56 ± 10.1	48.25 ± 14.93	**0.001**	68.6 ± 6.5	48.3 ± 14.9	** < 0.001**	** < 0.001**	1.000
Dealing with pain	36.11 ± 9.16	45.62 ± 17.11	**0.048**	63.3 ± 8.4	45.6 ± 17.1	** < 0.001**	** < 0.001**	1.000
Feeling of anxiety, fearing	34.2 ± 8.9	47.7 ± 14.1	**0.002**	66.6 ± 10.9	47.75 ± 14.1	** < 0.001**	** < 0.001**	1.000
Feeling of suffering from complications	32.7 ± 9.5	40 ± 16.3	0.121	62.7 ± 13.6	40 ± 16.3	** < 0.001**	** < 0.001**	1.000
Feeling Problems due to suffering sickle cell	32.7 ± 9.5	38.7 ± 17.08	0.211	61.1 ± 8.3	38.7 ± 17.1	** < 0.001**	** < 0.001**	1.000
Problems of treatment	41.1 ± 10.5	53.5 ± 13.2	**0.005**	63.7 ± 8.6	53.5 ± 13.3	**0.012**	** < 0.001**	1.000
Communications with others	39.6 ± 14.4	55.0 ± 11.5	**0.002**	62.2 ± 6.4	55 ± 11.5	**0.029**	** < 0.001**	1.000
Total score	35.7 ± 7.1	47.1 ± 10.4	**0.001**	64.9 ± 5.6	47.1 ± 10.4	** < 0.001**	** < 0.001**	1.000
Total children score)	39.8 ± 10.9	44.2 ± 6.9	0.194	70.2 ± 6.3	46.3 ± 8.3	** < 0.001**	** < 0.001**	0.300
Total parents score	35.7 ± 7.1	47.1 ± 10.4	**0.001**	64.9 ± 5.6	47.1 ± 10.4	** < 0.001**	** < 0.001**	0.326

**Fig. 4:**
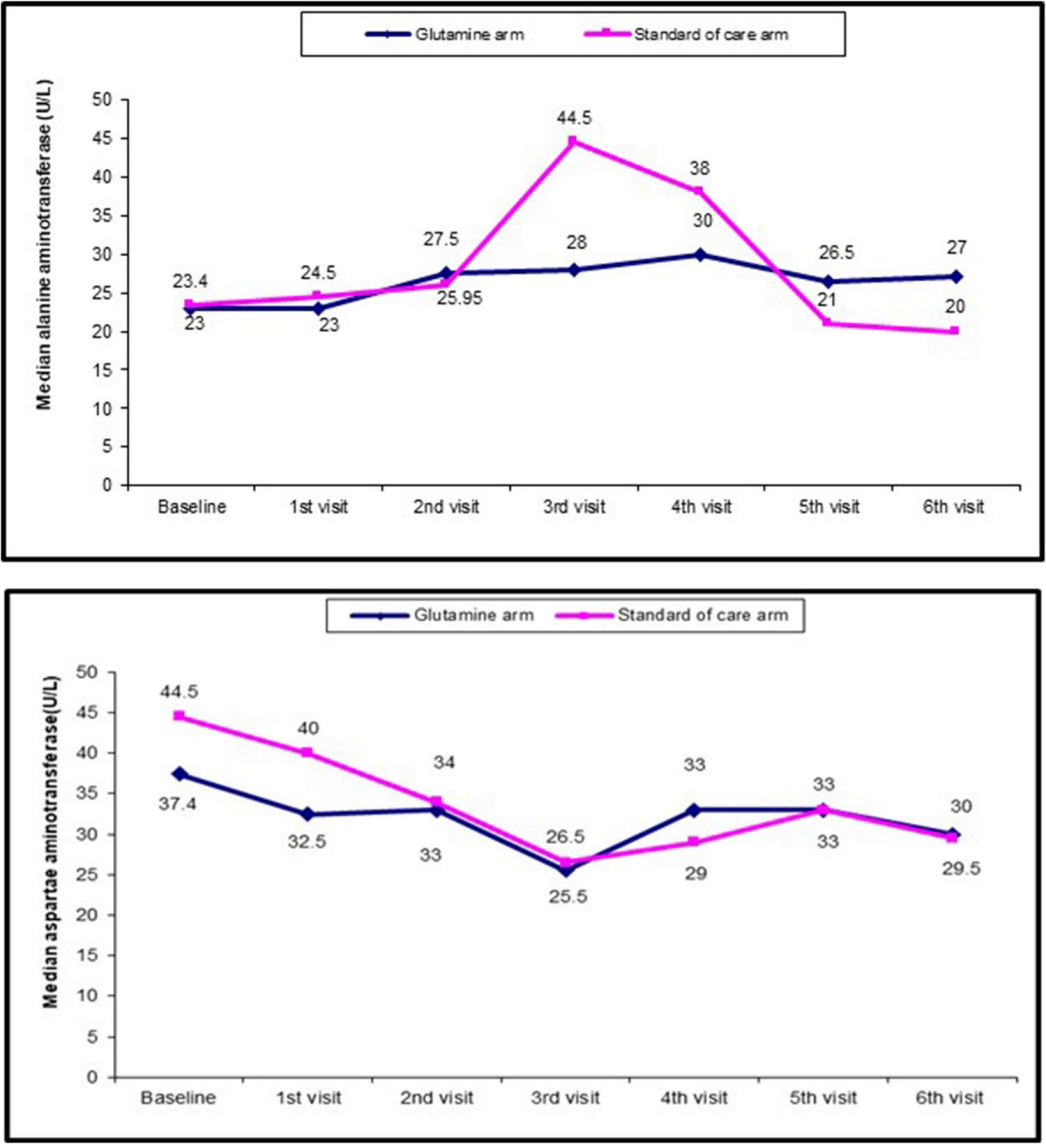
relation between alanine aminotransferase (above) and aspartame aminotransferase (below) of sixty patients through the six months of follow-up

As regards safety, five patients developed nausea, mild abdominal pain, and loose stool starting at week four of l-glutamine, and another four patients developed nausea, mild abdominal pain, a loose stool at week 12, and all resolved spontaneously. One serious adverse event (SAE) was reported, one patient after 12 weeks of treatment with l glutamine, presented with disturbed consciousness level, fever, vomiting, right upper quadrant abdominal pain, darkening of his urine with a deepening of his jaundice, and tachypnea, elevated transaminases tenfold with direct hyperbilirubinemia, derangement of kidney functions with abnormal bleeding profile, and metabolic acidosis with a positive anion gap, patient was diagnosed as sickle cell hepatopathy (SCH), admitted to PICU and was managed with supportive measures together, with exchange transfusion. The patient’s general condition improved with regaining full consciousness level and improvement of his labs and was planned for regular exchange transfusion, he stopped l-glutamine and was withdrawn from the study. As regards compliance, all patients were compliant and needed no change of dose of glutamine and only one patient withdrew from the study due to SAE. The ALT was significantly higher in the glutamine arm (p = 0.000) at week 12, this is due to the patient who developed SCH and was statistically significantly higher at week 24 (p < 0.001) but non-clinically significant as all values were within normal range. The serum creatinine was statistically significantly higher at week 24 (p = 0.002) but non-clinically significant as all values were within the normal range (Table 2 and Fig. 4).

A correlation study regarding questionnaire results of children and their parents at baseline in the glutamine arm (Table S1) showed that the age at diagnosis was positively correlated with how the child dealt with pain (p = 0.047), and feeling anger (p = 0.018), and disease duration was negatively correlated with most of the items of the assessment. The number of VOCs in last year correlated negatively with parental feelings of anxiety, fear (p = 0.024), feeling of suffering from complications (p = 0.003), feeling problems due to suffering sickle cell (p = 0.009), while the mean duration of VOC correlated negatively with child pain (p = 0.002), effects of pain (p = 0.002), how to deal with your pain (p = 0.022), anxiety problems with pain (p = 0.028), parental effects of pain (p = 0.035), feeling of anxiety, fearing (p = 0.010), feeling of suffering from complications (p = 0.008), feeling problems due to suffering sickle cell (p = 0.015), parents total score (p = 0.011). Correlation study regarding questionnaire results of children and their parents at baseline in SOC group illustrated in Table S2.

## Discussion

Over the last 50 years, there have been great improvements in the management of SCD. Until 2017, treatment options for SCD largely consisted of RBC transfusion [16], iron chelation [17], and HU [18, 19]. The story of l-glutamine as a proposed treatment for SCA started years ago, remarkably in the 1970s when there was a great interest in finding nontoxic “antisickling” agents [20]. In the 1980s, the finding that there is a decreased ratio of reduced NAD to total NAD in sickle RBCs compared with normal ones was discovered [21], and in the 1990s with the breakthrough discovery that there is a marked increased glutamine transport into sickle RBCs and ongoing oxidative injury support increased NAD synthesis and decreased NAD redox potential [22]*.*

The l-glutamine, the second drug used to reduce acute complications of SCD, was approved by the FDA in 2017 for use in patients over age 5 based on a phase 3 trial [7] The study by Niihara et al., recruited 230 patients from participating sites across the United States only and children aged from five to 12 years old representing only 22.4% [7], our study design to assess the safety and efficacy of L-glutamine in patients with SCD in a younger age group who all of them below 18 and in the different ethnic background to ensure the diversity of the result.

As 95% of patients with SCD get hospitalized due to severe episodes of pain or VOCs [23] and alleviating VOCs reduces both the frequency and duration of hospitalizations [24], our study aims to assess the effect of l-glutamine on in reducing the frequency of VOCs. In addition, our study evaluated the percentage of change in TCD- TAMMV- arterial cerebral blood flow, and its effect on improving HRQOL.

L-glutamine is a conditionally essential amino acid in SCD due to the high RBC turnover [25], and its therapeutic mechanism is mainly through its antioxidant effects [7]. Glutamine suppresses several inflammatory signaling pathways, including suppressing nuclear factor-κB (NFκB) signaling pathway [26] and signal transducer and activator of transcription (STAT) pathways [27], by increasing the expression of cellular HSPs such as HSP25 and HSP70 [28] and inhibition of the expression of IL-8 which activates and stimulates the migration of neutrophils to inflammatory sites [29].

In our study, sixty participants were recruited; their mean age was around nine years old, and all of them were on HU stable dose at enrollment, as long-term use of HU is safe and well-tolerated in large cohorts of children and adults with SCD, reducing mortality and morbidity of both children and adult patients [30, 31].

At baseline, all patients in both arms have had VOCs in the last year more than three; patients on glutamine had a higher number of VOCs and hospitalization for VOCs in the last year. However, after the start of one month of the glutamine, there was a significant improvement in the severity of VOCs and number of hospitalizations for VOCs, this improvement continues through the treatment period. After 24 weeks of treatment with glutamine, there was a significant decrease in the number and severity of VOCs and hospitalization of VOCs in the glutamine arm.

Several studies prove the effect of l-glutamine on reducing the frequency of VOCs; a randomized controlled trial recruited 81 SCD patients aged from 9 to 58 years, who were randomized 1:1 to glutamine 0.3 g/kg orally twice daily or placebo for 48 weeks showed that l-glutamine therapy reduced the frequency of hospitalization and indicated a positive trend towards reducing the frequency of painful crises [10], also results of a phase 3 trial showed that treatment with l-glutamine led to a statistically significant reduction in the frequency of pain crises and rates of hospitalization [7], Elenga and his colleagues reported that SCD patients, whom nearly half of them receiving HU simultaneously (47%), demonstrated a clinically significant reduction in the median frequency of VOCs at 24-, 48- and 72-weeks following L-glutamine therapy compared to the preceding year [32].

In our study, after 24 weeks of treatment, L-glutamine did not cause a reduction in hemolysis markers nor improvement of hemoglobin level, as there was no difference between the two groups except at week 12, the reticulocyte count was higher in those on SOC. This is in agreement with a phase 3 study [7] in that neither the Hb nor the reticulocyte counts differed significantly between the group assigned to L-glutamine and placebo. Cox et al. reported there is no evidence currently that hemoglobin increases on l-glutamine or that hemolysis is reduced [6]. On the other side for the first time, Elenga et al. demonstrated significant improvement in clinical parameters along with improvement in hemolysis parameters as regards reticulocyte count, LDH, Hemoglobin level, and hematocrit level [32].

In children, cerebral vasculopathy is the most common CNS complication resulting from progressive inflammation and oxidative endothelial damage within intracranial vessels, with the influence of some genetic modifiers [33], leading to increased risk of transient ischemic events and infarcted strokes [34] and long term neurocognitive and psychiatric problems [35]. Screening for vasculopathy beginning at age two is recommended [36] in the form of TCD measurements which are effective in identifying children with SCA at the highest risk for stroke [37]. In our cohort, TCD parameters at baseline and follow-up in the glutamine arm, there were four patients in the glutamine arm below 70cm/sec in the left MCA at baseline, dropped to three patients at week 12 and none at week 24. Although l-glutamate is the most abundant amino acid in the brain and a key neurotransmitter involved in the maintenance of brain health and plasticity [38] under chronic oxidative stress, levels of deoxygenated hemoglobin in the brain may increase with increasing GSH depletion, affecting brain microstructure and cognitive function [39], no published study addresses the effect of L-glutamine on TCD measurements.

The glutamine role in the quality of life or other patient-reported outcomes needs to be examined and should not be overlooked [40] we reported that in patients who received glutamine, there was a significant improvement in pain, how they dealt with pain, anxiety, anger and treatment problems for children and parents of children aged 5–7 years and age 8–12 years.

Most of the adverse events in our cohort were nausea, mild abdominal pain, and loose stool which resolved spontaneously, this is in agreement with the previous study [7]. Glutamine is an important substrate for rapidly dividing cells, for instance, RBCs, which is a major site of glutamine consumption [41]. Intestinal pathophysiologic changes that may be modified by glutamine in SCD include increased intestinal permeability and increased translocation of bacteria into the systemic circulation promoting ongoing inflammatory processes that activate blood cells and platelets involved in the development of VOC. Glutamine may have a role in improving intestinal health, fortifying intestinal barrier functions, modifying microbial compositions, and decreasing inflammatory processes [42]. Glutamine may modify the intestinal microbial community [43], it reduces the abundance of *Clostridium* spp*.* in the ileum and *Helicobacter* spp*.* in the ileum and cecum [44] this may be related to nitrogen balance and protein synthesis by the *Firmicutes* spp. residing in the small intestine [45].

One SAE reported after 12 weeks of treatment with l glutamine as sickle cell hepatopathy (SCH) completely recovered and withdrawn from the study. At the end of the study, ALT and serum creatinine were statistically significantly higher but non-clinically significant as all values were within the normal range. There is no long-term data about glutamine use in SCD who are at risk of renal insufficiency, hepatic dysfunction, and multiorgan failure, all conditions associated with increased mortality in other trials of l-glutamine [20].

Challenges of usage of glutamine, it is much more expensive and naturalistic, so it is reasonable to consider its usage only after the optimization of HU therapy and not at the risk of reducing adherence to HU and use it as a primary therapy for those who tolerate HU poorly [20].

## Conclusion

L-glutamine appears to be reasonably well tolerated in the short term, and effectively reduced the number of VOCs and hospitalizations for VOC and might have a potential favorable impact on the cerebral arterial flow velocities. However, larger long-term studies are needed.

### Supplementary Information

Below is the link to the electronic supplementary material.

Supplementary file1 (JPG 170 KB)

Supplementary file2 (JPG 128 KB)

Supplementary file3 (DOCX 31 KB)

## Data Availability

No datasets were generated or analysed during the current study.
