# Supplementary figures and images for: Safety and efficacy of L-Glutamine in reducing the frequency of acute complications among patients with sickle cell disease: A randomized controlled study
Source: Ann Hematol. 2024 Jul 19;103(9):3493–506. doi: 10.1007/s00277-024-05877-8 (PMC11358349; doi:10.1007/s00277-024-05877-8)

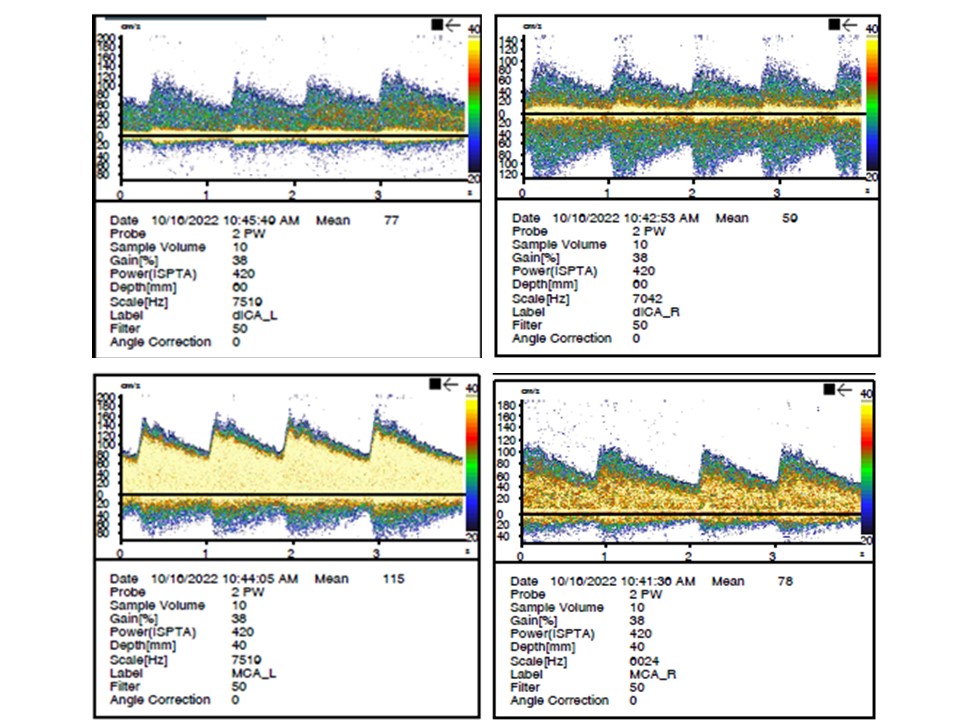

Supplement: Supplementary file 1 — Supplementary file1 (JPG 170 KB) [file 277_2024_5877_MOESM1_ESM.jpg]

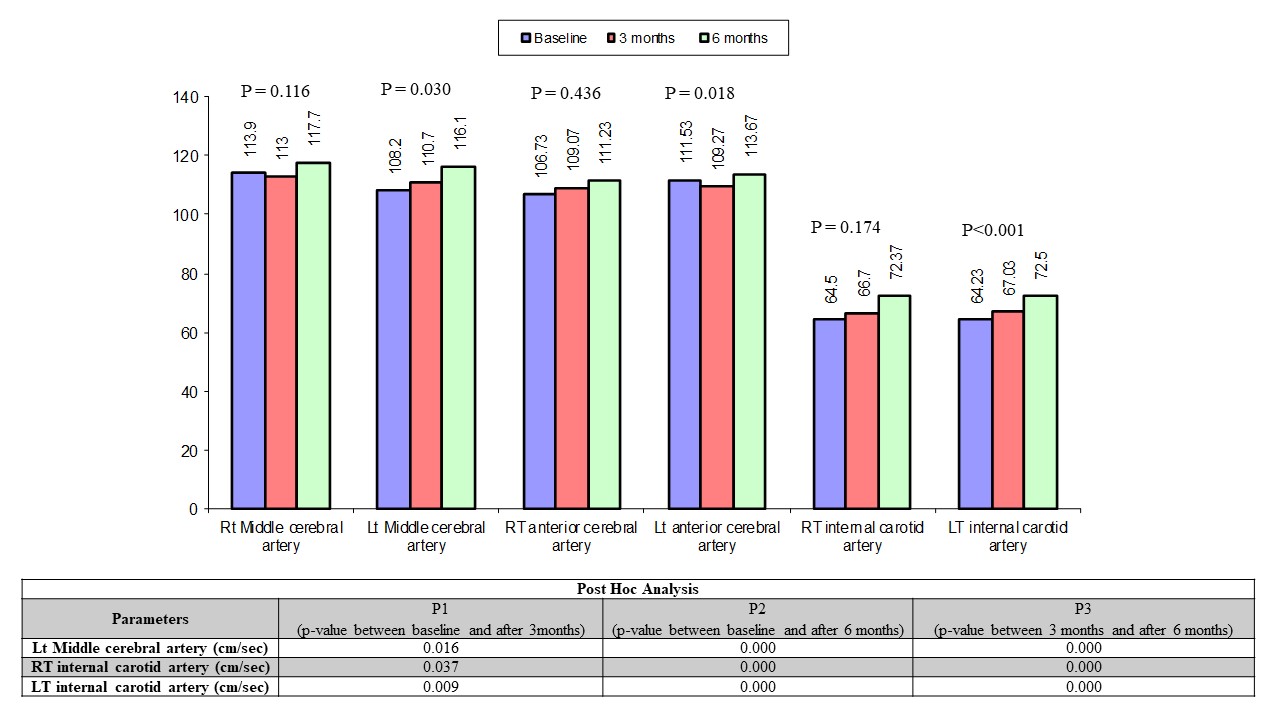

Supplement: Supplementary file 2 — Supplementary file2 (JPG 128 KB) [file 277_2024_5877_MOESM2_ESM.jpg]
