# Supplementary material for: Safety and efficacy of L-Glutamine in reducing the frequency of acute complications among patients with sickle cell disease: A randomized controlled study
Source: Ann Hematol. 2024 Jul 19;103(9):3493–506. doi: 10.1007/s00277-024-05877-8 (PMC11358349; doi:10.1007/s00277-024-05877-8)
Supplement: Supplementary file 3 — Supplementary file3 (DOCX 31 KB) [file 277_2024_5877_MOESM3_ESM.docx]

**Table S1:** Correlation of age of diagnosis, disease duration, number of vaso-occlusion crisis last year and mean duration of vaso-occlusion crisis with questionnaire results of children and their parents at baseline in glutamine arm (intervention group).

|  | **Age of diagnosis  in months** | | **Disease  Duration (Years)** | | **Number of  vaso-occlusion crisis/ Last year** | | **Mean Duration  of vaso-occlusion crisis (days)** | |
| --- | --- | --- | --- | --- | --- | --- | --- | --- |
|  | **r** | **p-value** | **R** | **p-value** | **r** | **p-value** | **R** | **p-value** |
| My pain | 0.227 | 0.228 | **-0.782**** | **<0.001** | -0.214 | 0.256 | **-0.537**** | **0.002** |
| Effects of pain | 0.166 | 0.381 | **-0.595**** | **0.001** | -0.265 | 0.156 | **-0.534**** | **0.002** |
| How to deal with your pain | **0.366*** | **0.047** | **-0.448*** | **0.013** | -0.261 | 0.163 | **-0.418*** | **0.022** |
| Anxiety problems with pain | 0.147 | 0.438 | **-0.403*** | **0.027** | -0.289 | 0.121 | **-0.401*** | **0.028** |
| Angry feeling | **0.428*** | **0.018** | 0.090 | 0.635 | -0.299 | 0.108 | 0.094 | 0.621 |
| Treatment problems | 0.263 | 0.159 | **-0.443*** | **0.014** | 0.179 | 0.344 | -0.291 | 0.118 |
| Communications problems | 0.000 | 0.999 | -0.302 | 0.105 | 0.005 | 0.980 | -0.355 | 0.054 |
| **Children total score** | 0.142 | 0.454 | **-0.522**** | **0.003** | -0.285 | 0.127 | **-0.448*** | **0.013** |
| Feeling of pain | 0.277 | 0.139 | **-0.628**** | **<0.001** | -0.288 | 0.123 | -0.344 | 0.063 |
| Effects of pain | -0.094 | 0.621 | -0.358 | 0.052 | -0.292 | 0.117 | **-0.386*** | **0.035** |
| Dealing with pain | 0.135 | 0.477 | -0.337 | 0.069 | -0.211 | 0.264 | -0.264 | 0.159 |
| Feeling of anxiety, fearing | -0.148 | 0.436 | -0.239 | 0.204 | **-0.412*** | **0.024** | **-0.461*** | **0.010** |
| Feeling of suffering from complications | 0.185 | 0.329 | **-0.462*** | **0.010** | **-0.523**** | **0.003** | **-0.474**** | **0.008** |
| Feeling Problems due to suffering sickle cell | -0.052 | 0.784 | -0.277 | 0.139 | **-0.467**** | **0.009** | **-0.441*** | **0.015** |
| Problems of treatment | 0.206 | 0.276 | **-0.447*** | **0.013** | 0.195 | 0.301 | 0.067 | 0.723 |
| Communications with others | -0.021 | 0.911 | **-0.382*** | **0.037** | 0.146 | 0.442 | -0.238 | 0.205 |
| **Parents total score** | -0.087 | 0.646 | **-0.671**** | **<0.001** | -0.329 | 0.076 | **-0.459*** | **0.011** |

**Table S2:** Correlation of age of diagnosis, disease duration, number of vaso-occlusion crisis last year and mean duration of vaso-occlusion crisis with questionnaire results of children and their parents at baseline in standard of care arm.

|  | **Age of diagnosis  in months** | | **Disease  duration (Years)** | | **Number of  vaso-occlusion crisis / last year** | | **Mean Duration  of vaso-occlusion crisis (days)** | |
| --- | --- | --- | --- | --- | --- | --- | --- | --- |
|  | **R** | **p-value** | **R** | **p-value** | **R** | **p-value** | **R** | **p-value** |
| My pain | 0.075 | 0.693 | **-0.502**** | **0.005** | **-0.478**** | **0.008** | -0.203 | 0.282 |
| Effects of pain | **0.482**** | **0.007** | **-0.494**** | **0.006** | -0.078 | 0.681 | 0.051 | 0.790 |
| How to deal with your pain | **0.451*** | **0.012** | **-0.485**** | **0.007** | -0.248 | 0.186 | -0.227 | 0.227 |
| Anxiety problems win | 0.128 | 0.501 | **-0.618**** | **0.000** | **-0.492**** | **0.006** | **-0.440*** | **0.015** |
| Angry feeling | 0.161 | 0.395 | -0.022 | 0.907 | -0.337 | 0.068 | -0.032 | 0.865 |
| Treatment problems | 0.114 | 0.549 | -0.011 | 0.956 | -0.001 | 0.996 | -0.157 | 0.408 |
| Communications problems | 0.188 | 0.320 | 0.058 | 0.761 | 0.039 | 0.838 | -0.021 | 0.912 |
| **Children total score** | 0.260 | 0.166 | -0.437* | 0.016 | -0.308 | 0.097 | -0.217 | 0.250 |
| Feeling of pain | 0.113 | 0.551 | **-0.365*** | **0.047** | **-0.415*** | **0.022** | -0.354 | 0.055 |
| Effects of pain | -0.126 | 0.509 | 0.132 | 0.486 | **-0.378*** | **0.039** | -0.192 | 0.310 |
| Dealing with pain | -0.018 | 0.925 | -0.132 | 0.486 | -0.341 | 0.065 | -0.230 | 0.221 |
| Feeling of anxiety, fearing | 0.322 | 0.083 | 0.058 | 0.761 | -0.288 | 0.122 | 0.214 | 0.256 |
| Feeling of suffering from complications | 0.240 | 0.202 | 0.008 | 0.968 | -0.143 | 0.450 | 0.171 | 0.367 |
| Feeling Problems due to suffering sickle cell | 0.199 | 0.291 | 0.016 | 0.932 | -0.141 | 0.456 | 0.113 | 0.552 |
| Problems of treatment | 0.002 | 0.992 | 0.082 | 0.668 | 0.146 | 0.442 | -0.072 | 0.704 |
| Communications with others | **0.382*** | **0.037** | -0.165 | 0.385 | 0.108 | 0.571 | -0.037 | 0.847 |
| **Parents total score** | 0.202 | 0.283 | 0.043 | 0.821 | -0.205 | 0.278 | -0.025 | 0.896 |
